# Revisiting the Geriatric Depression Scale: An IRT-Based 10-Item Screen Outperforms the GDS-15 in Diagnostic Accuracy and Efficiency

**DOI:** 10.3390/jcm15020473

**Published:** 2026-01-07

**Authors:** Ji Won Han, Dae Jong Oh, Tae Hui Kim, Kyung Phil Kwak, Bong Jo Kim, Shin Gyeom Kim, Jeong Lan Kim, Seok Woo Moon, Joon Hyuk Park, Seung-Ho Ryu, Jong Chul Youn, Dong Young Lee, Dong Woo Lee, Seok Bum Lee, Jung Jae Lee, Jin Hyeong Jhoo, Ki Woong Kim

**Affiliations:** 1 Department of Neuropsychiatry, Seoul National University Bundang Hospital, 82 Gumi-ro 173-beon-gil, Bundang-gu, Seongnam 13620, Republic of Korea; jwhanmd@snu.ac.kr; 2 Department of Psychiatry, Seoul National University College of Medicine, Seoul 08826, Republic of Korea; selfpsy@snu.ac.kr; 3 Workplace Mental Health Institute, Kangbuk Samsung Hospital, Sungkyunkwan University School of Medicine, Seoul 06351, Republic of Korea; pauloh0423@gmail.com; 4 Department of Psychiatry, Yonsei University Wonju Severance Christian Hospital, Wonju 26426, Republic of Korea; gooddr@yonsei.ac.kr; 5 Department of Psychiatry, Dongguk University Gyeongju Hospital, Gyeongju 38067, Republic of Korea; drphil@dongguk.ac.kr; 6 Department of Psychiatry, Gyeongsang National University School of Medicine, Jinju 52727, Republic of Korea; bjkim@gnu.ac.kr; 7 Department of Neuropsychiatry, Soonchunhyang University Bucheon Hospital, Bucheon 14584, Republic of Korea; redmensch@schmc.ac.kr; 8 Department of Psychiatry, School of Medicine, Chungnam National University, Daejeon 34134, Republic of Korea; kimjl@cnu.ac.kr; 9 Department of Psychiatry and Research Institute of Medical Science, Konkuk University, Konkuk University Chungju Hospital, Chungju 27478, Republic of Korea; hessem@kku.ac.kr; 10 Department of Neuropsychiatry, Jeju National University Hospital, Jeju 63243, Republic of Korea; empath0125@gmail.com; 11 Department of Psychiatry, School of Medicine, Konkuk University Medical Center, Konkuk University, Seoul 05029, Republic of Korea; shryu@kuh.ac.kr; 12 Department of Neuropsychiatry, Kyunggi Provincial Hospital for the Elderly, Yongin 36114, Republic of Korea; yjc0811@naver.com; 13 Department of Neuropsychiatry, Seoul National University Hospital, Seoul 03080, Republic of Korea; 14 Department of Neuropsychiatry, Inje University Sanggye Paik Hospital, Seoul 01757, Republic of Korea; dwlee@paik.ac.kr; 15 Department of Psychiatry, Dankook University Hospital, Cheonan 31116, Republic of Korea; bumlee@dankook.ac.kr (S.B.L.); mdjjlee@gmail.com (J.J.L.); 16 Department of Psychiatry, College of Medicine, Kangwon National University, Chuncheon 24341, Republic of Korea; jhoojh@kangwon.ac.kr; 17 Department of Brain and Cognitive Science, Seoul National University College of Natural Sciences, Seoul 08826, Republic of Korea; 18 Institute of Human Behavioral Medicine, Seoul National University Medical Research Center, Seoul 03080, Republic of Korea

**Keywords:** depression, screening, item response theory, geriatric depression scale, psychometrics

## Abstract

**Background/Objective**: Existing abbreviated Geriatric Depression Scales (GDSs), derived via Classical Test Theory (CTT), often sacrifice accuracy for brevity and retain non-specific items. We aimed to develop a minimum-item GDS maintaining diagnostic performance equivalent to the full 30-item scale (GDS30) using Item Response Theory (IRT). **Methods**: This cross-sectional study employed rigorous 5:5 split-sample cross-validation. Participants included 6525 older adults (aged ≥60 years) from community-based (Korean Longitudinal Study on Cognitive Aging and Dementia) and clinical settings (geropsychiatry clinic). Depression was diagnosed through standardized clinical interviews based on DSM-IV criteria. Two-parameter logistic IRT models estimated item discrimination and difficulty parameters. Sequential item reduction with DeLong tests identified the minimum number of items required to maintain GDS30-equivalent area under the curve (AUC). **Results**: The 10-item IRT-optimized scale (GDS10-IRT) achieved an AUC of 0.856 (95% CI: 0.809–0.895) in the validation set, showing no significant difference from GDS30 (AUC = 0.883; *p* = 0.396). Conversely, the 15-item GDS (GDS15) demonstrated significantly lower AUC than GDS30 (*p* < 0.001) despite having more items. GDS10-IRT achieved a 234% improvement in efficiency ratio (AUC/items) over GDS30. Notably, Item 16 (“feeling downhearted and blue”), identified as the most discriminating symptom (a = 2.53), is absent from the GDS15 but included in GDS10-IRT. **Conclusions**: IRT-based item selection achieves GDS30-equivalent diagnostic accuracy with only 10 items, outperforming the widely used GDS15. By recovering high-discrimination items excluded by CTT, the GDS10-IRT offers a more efficient, specific screening tool for late-life depression.

## 1. Introduction

Late-life depression (LLD) is one of the most prevalent psychiatric disorders among older adults and is associated with increased morbidity, mortality, medical illness, and dementia [1]. Depression is the leading cause of disability measured by Years Lived with Disability and the fourth leading contributor to the global burden of disease [2]. However, LLD remains underrecognized and undertreated due to its subsyndromal features and complicated etiologies.

The 30-item Geriatric Depression Scale (GDS30), developed by Yesavage and colleagues, has become one of the most widely used depression screening instruments for older adults [3]. Unlike other screening instruments such as the Beck Depression Inventory [4] or the Center for Epidemiologic Studies Depression Scale [5], the GDS does not contain items regarding physical symptoms that are prevalent in older adults due to comorbid medical conditions. Instead, it uses a simple yes/no response format that enhances reliability and shortens administration time in elderly populations.

Despite these advantages, the length of GDS30 poses practical barriers in busy clinical settings, prompting the development of numerous abbreviated versions. The most widely adopted short form is the 15-item GDS (GDS15), developed using Classical Test Theory (CTT) methods on Western samples [6]. Subsequently, even shorter versions have been proposed, including a 4-item version [7] and a 10-item version [8]. However, all these abbreviated versions were derived from GDS15 using CTT methods, inheriting any limitations in its item selection.

CTT selects items based on item-total correlations, which favor moderately endorsed items while potentially overlooking items with low endorsement rates but high diagnostic specificity. Item Response Theory (IRT) offers a fundamentally different approach, estimating each item’s discrimination power and difficulty independently of endorsement frequency [9]. This allows identification of ‘quiet but powerful’ items (symptoms rarely reported but almost diagnostic when present) that CTT methods may systematically exclude.

A critical but often overlooked question in scale abbreviation is: how many items are actually necessary to maintain the full scale’s diagnostic accuracy? The widespread acceptance of 15- and 10-item versions assumes that significant item reduction necessarily compromises performance. However, if IRT can identify the most discriminating items from the complete item pool, substantially fewer items might suffice while maintaining or even improving diagnostic accuracy.

We hypothesized that IRT-based item selection from the complete GDS30 item pool would identify a minimum-item version that: (1) maintains diagnostic accuracy statistically equivalent to GDS30, (2) requires fewer items than existing CTT-derived short forms, and (3) recovers high-discrimination items that were lost in previous CTT-based reductions. Specifically, we suspected that the widely accepted 15-item threshold might be an artifact of CTT methodology rather than a true psychometric necessity. This study aimed to develop and validate the minimum-item GDS maintaining GDS30 equivalent performance, with rigorous cross-validation to ensure generalizability.

## 2. Methods

### 2.1. Study Design and Participants

Data were drawn from the Korean Longitudinal Study on Cognitive Aging and Dementia (KLOSCAD), an ongoing nationwide population-based prospective cohort study [10,11], and clinical samples from the Geropsychiatry Clinic of Seoul National University Bundang Hospital. KLOSCAD participants were randomly sampled from residents aged 60 years or older in 13 districts across South Korea, stratified by age and sex. The total sample comprised 6525 participants (community-dwelling: n = 5872; clinical: n = 653).

All participants provided written informed consent, and the study protocol was approved by the Institutional Review Board of Seoul National University Bundang Hospital.

### 2.2. Diagnostic Assessment

Standardized clinical interviews, physical examinations, and neurological examinations were administered to all participants using the Korean version of the Mini-International Neuropsychiatric Interview (MINI) [12] and the Korean version of the Consortium to Establish a Registry for Alzheimer’s Disease assessment battery (CERAD-K) [13] by geropsychiatrists with advanced training in geriatric psychiatry and dementia research. Axis I psychiatric disorders including major and minor depressive disorder were diagnosed according to the fourth edition of the Diagnostic and Statistical Manual of Mental Disorders (DSM-IV) criteria [14]. Participants with dementia, delirium, or other major psychiatric disorders that could affect mood assessment were excluded.

### 2.3. Geriatric Depression Scale

All participants completed the GDS30, which was developed through rigorous translation and back-translation procedures and has demonstrated excellent psychometric properties in Korean older adults (Cronbach’s α = 0.90; test–retest reliability = 0.91) [15].

### 2.4. Sample Splitting for Cross-Validation

To ensure robust validation and prevent overfitting, the sample was randomly divided into development (n = 3262) and validation (n = 3263) sets using 5:5 stratified sampling by depression status and enrollment source. IRT parameters were estimated in the development set, and diagnostic accuracy was evaluated in both sets independently. Cross-validation was assessed by comparing the area under the receiver operating characteristics (ROC) curves between development and validation sets for each scale using DeLong tests [16]; non-significant differences (*p* > 0.05) would indicate stable performance without overfitting.

### 2.5. Item Response Theory Analysis

Two-parameter logistic (2PL) IRT models were fit to all 30 GDS items in the development set using the mirt package in R (version 4.2.0). For each item, two parameters were estimated: (1) the discrimination parameter (*a*), indicating how well the item differentiates between depressed and non-depressed individuals; and (2) the difficulty parameter (*b*), indicating the depression severity level at which the item has a 50% probability of being endorsed. Items were ranked by discrimination, with the highest-discriminating items selected for the short form.

### 2.6. Sequential Item Reduction Analysis

To determine the minimum number of items maintaining GDS30 equivalent diagnostic performance, we performed sequential item reduction analysis. Starting from the top 10 discriminating items, we progressively removed items one at a time in order of lowest discrimination. At each reduction step, we calculated the AUC in both development and validation sets and compared each against GDS30 using the DeLong test [16]. The minimum-item version was defined as the fewest number of items showing no statistically significant AUC difference from GDS30 (*p* > 0.05) in the validation set, with the additional requirement of non-significance in the development set to ensure stability. This dual-criterion stopping rule guards against chance findings that might not replicate.

Rather than arbitrarily selecting a round number of items (e.g., 10 or 15), we employed a data-driven approach to determine the optimal scale length. Sequential reduction was performed by removing items one at a time in reverse order of discrimination. At each reduction step, DeLong tests compared the abbreviated scale’s AUC against GDS30. The optimal number of items was defined as the minimum that maintained statistical equivalence to GDS30 (*p* > 0.05) in the development set. Additionally, we examined the test information function to identify potential ‘elbow points’ where marginal information gains diminished substantially.

### 2.7. Statistical Analysis

Demographic and clinical characteristics were summarized using means and standard deviations (SD) for continuous variables and frequencies and percentages for categorical variables. Comparisons between development and validation sets were performed using independent samples *t*-tests for continuous variables and chi-square tests for categorical variables to confirm successful randomization.

ROC curve analyses were performed to evaluate diagnostic accuracy. AUC comparisons between scales were performed using the DeLong test for correlated ROC curves. Sensitivity and specificity were calculated at optimal cutoff scores determined by Youden’s index. Comparisons of sensitivity and specificity between scales within the same sample were performed using McNemar’s test for paired proportions, while comparisons between development and validation sets were performed using chi-square tests for independent proportions.

The efficiency ratio was defined as AUC divided by the number of items (AUC/items), quantifying diagnostic accuracy achieved per item. To compare efficiency ratios between scales within the same sample, we employed a bootstrap approach: in each of 1000 bootstrap iterations (the standard for bootstrap inference), we resampled subjects with replacement, calculated AUCs for both scales, computed the efficiency ratio for each, and obtained the difference. Statistical significance was determined using the percentile method; if the 95% bootstrap confidence interval for the difference excluded zero, the comparison was considered significant (*p* < 0.05). For cross-validation comparisons (development vs. validation sets), efficiency ratios and their bootstrap standard errors were computed independently in each set. The difference was tested using a z-statistic: *z* = (*Efficiency_dev_* − *Efficiency_val_*)/√(*SE*^2^*_dev_* + *SE*^2^*_val_*), with significance assessed against the standard normal distribution.

To evaluate measurement invariance, we examined differential item functioning (DIF) across three grouping variables: sex (male vs. female), age group (young-old [60–74 years] vs. old-old [≥75 years]), and recruitment setting (community vs. clinical). DIF was assessed using the Mantel-Haenszel (MH) procedure, with effect sizes classified according to Educational Testing Service (ETS) criteria: Category A (|ΔMH| < 1.0; negligible), Category B (1.0 ≤ |ΔMH| < 1.5 with *p* < 0.05; moderate), and Category C (|ΔMH| ≥ 1.5 with *p* < 0.05; large). To assess scale-level impact (Differential Test Functioning), we compared AUC values across subgroups.

All statistical analyses were performed using R (version 4.5.2). IRT analysis was conducted using the mirt package for two-parameter logistic model estimation. ROC curve analyses, including AUC calculation with 95% confidence intervals and DeLong tests for correlated ROC curves, were performed using the pROC package. Internal consistency (Cronbach’s α) was calculated using the psych package. Statistical significance was set at α = 0.05 (two-tailed).

## 3. Results

### 3.1. Sample Characteristics

Table 1 presents the demographic and clinical characteristics of the total sample and comparisons between development and validation sets. The total sample (N = 6525) had a mean age of 72.4 years (SD = 7.2; range: 60–95) and was 58.0% female. Mean education was 7.8 years (SD = 5.2). The majority of participants were enrolled from the KLOSCAD, with the remaining 10.0% recruited from the clinic. All participants were community-dwelling. Depression prevalence was 3.8% overall (n = 248), with higher rates in clinic sample (13.0%) compared to the KLOSCAD sample (3.2%). The development (n = 3262) and validation (n = 3263) sets showed no significant differences in age, sex, education, recruitment source, GDS30 total score, or depression prevalence (all *p* > 0.05), confirming successful randomization.

### 3.2. IRT Item Parameters and Cross-Version Comparison

Table 2 presents the IRT parameters for all 30 GDS items along with their inclusion status across four short form versions. Item 16 (‘Do you often feel downhearted and blue?’) demonstrated the highest discrimination (*a* = 2.53) and individual-item AUC (0.779) among all 30 items, yet it is conspicuously absent from GDS15 and all its derivative short forms. Notably, Item 16’s difficulty parameter (*b* = 0.60) indicates moderate endorsement frequency—not a rare symptom—suggesting its exclusion from GDS15 was likely due to perceived redundancy with other mood items rather than low endorsement. IRT analysis reveals this apparent ‘redundancy’ actually reflects Item 16’s role as a diagnostic anchor that maximally discriminates between depressed and non-depressed individuals.

In contrast, GDS15 retains several items with notably low discrimination: Item 12 (‘prefer to stay at home’; *a* = 0.70), Item 14 (‘more memory problems than most’; *a* = 0.88), and Item 2 (‘dropped activities/interests’; *a* = 0.91). The mean discrimination of GDS15 items (*a* = 1.38) was 20% lower than that of GDS10-IRT items (*a* = 1.96), indicating that GDS10 comprises a more ‘elite’ set of high-performing items.

### 3.3. Sequential Item Reduction with Cross-Validation

Table 3 presents the sequential item reduction analysis with results from both development and validation sets, along with cross-validation statistics. The version including 10 items (GDS10-IRT) was identified as the minimum version maintaining GDS30 equivalent performance, showing non-significant differences from GDS30 in both development set (*p* = 0.576) sets. The cross-validation *p*-value (0.210) confirmed stable performance across samples without evidence of overfitting. At 9 items, statistically significant performance degradation occurred in the validation set (development: *p* = 0.003; validation: *p* = 0.298), confirming that 10 items represent the minimum threshold for GDS30 equivalence. While the 9-item version showed non-significant difference in the validation set (*p* = 0.298), the significant degradation in the development set (*p* = 0.003) indicated instability that could compromise generalizability, establishing 10 items as the robust minimum. The 10th-ranked item by discrimination was Item 6 (‘feeling that life is empty’; a = 1.71). The test information function revealed an elbow point at 10 items: the marginal decrease in information from 10 to 9 items (Δ = 0.12) was substantially larger than the decrease from 11 to 10 items (Δ = 0.08), indicating diminishing returns beyond 10 items.

A notable finding emerged for GDS15 was that its performance was significantly inferior to GDS30 in both the development (*p* = 0.012) and validation (*p* < 0.001) sets. This pattern suggests that GDS15’s marginal performance disadvantage becomes apparent with independent validation, underscoring the importance of cross-validation in scale development studies.

### 3.4. GDS10-IRT Item Composition

The final GDS10 comprises Items 1, 4, 6, 10, 11, 16, 17, 21, 22, and 25 (see Table 2 for details). Internal consistency was excellent (Cronbach’s α = 0.83). The optimal cutoff score was ≥4, yielding sensitivity of 80.0% and specificity of 84.0% in the validation set. Four items not included in GDS15 (Items 6, 11, 16, 25) capture core dysphoria and the anxiety/agitation dimensions prominent in late-life depression.

### 3.5. Screening Performance and Efficiency Comparison

Table 4 presents the comparison of sensitivity, specificity, and efficiency ratio across scales with statistical testing. Both GDS15 and GDS10-IRT showed sensitivity and specificity statistically equivalent to GDS30 (all McNemar *p* > 0.05), indicating that item reduction did not significantly compromise classification accuracy at optimal cutoffs.

However, the efficiency ratio defined as AUC per item differed substantially across scales. GDS10-IRT achieved an efficiency ratio of 0.085 in the validation set, significantly higher than both GDS30 (0.029; bootstrap *p* < 0.001) and GDS15 (0.057; bootstrap *p* < 0.001). This represents a 234% improvement over GDS30 and a 70% improvement over GDS15. Importantly, all metrics showed no significant differences between development and validation sets (all cross-validation *p* > 0.05), confirming stable performance without overfitting.

### 3.6. Differential Item Functioning

DIF analysis examined measurement invariance across sex, age group, and recruitment setting (Table 5). By sex, 4 of 10 items showed meaningful DIF: GDS06 (‘afraid something bad will happen’), GDS11 (‘wonderful to be alive’), GDS16 (‘downhearted and blue’), and GDS25 (‘feel like crying’) were more readily endorsed by women. Item 25 exhibited large DIF (ΔMH = 2.27, ETS Category C). By age group, 3 items showed meaningful DIF: GDS10 (‘memory problems’), GDS17 (‘feel worthless’), and GDS22 (‘situation is hopeless’) were more readily endorsed by older adults (≥75 years). By recruitment setting, 8 items showed DIF, with clinical participants endorsing items more readily than community participants. Despite item-level DIF, scale-level diagnostic performance (Differential Test Functioning) was equivalent across sex and age groups. AUC values were comparable between males (0.856; 95% CI: 0.786–0.925) and females (0.817; 95% CI: 0.778–0.856), with ΔAUC = 0.039. Similarly, young–old (0.839; 95% CI: 0.801–0.878) and old–old (0.832; 95% CI: 0.762–0.902) groups showed equivalent performance (ΔAUC = 0.007). The larger difference between community (0.861) and clinical (0.695) settings (ΔAUC = 0.166) reflects population differences in depression characteristics rather than measurement bias.

## 4. Discussion

In this study, we challenged the conventional assumption that more items necessarily yield better diagnostic accuracy in depression screening. By applying Item Response Theory to a large population-based Korean cohort with rigorous cross-validation, we demonstrated that 10 optimally selected items achieve diagnostic accuracy statistically equivalent to the full 30-item GDS, which is a finding with substantial implications for clinical practice and scale development methodology. The GDS10-IRT achieved an efficiency ratio of 0.097 (AUC/items) in the validation set, representing a 234% improvement over GDS30 (0.029) and a 70% improvement over GDS15 (0.057). Critically, all performance metrics showed no significant differences between development and validation sets (all cross-validation *p* > 0.05), confirming stable performance without overfitting.

### 4.1. Overcoming the Specificity Pitfalls of CTT-Based Short Forms

The superior efficiency of GDS10-IRT over GDS15 stems from its systematic exclusion of items that function as diagnostic ‘noise.’ A critical limitation shared by CTT-derived scales including the GDS15 and GDS10 is their retention of items based primarily on item-total correlations, which inherently favors moderately endorsed but non-specific symptoms over highly discriminating core features.

Specifically, existing CTT-based short forms retain items such as Item 14 (‘Do you feel you have more problems with memory than most?’) and Item 12 (‘Do you prefer to stay at home rather than going out and doing new things?’). As identified in our previous factor analytic study [15], these items load heavily on ‘Cognitive Inefficiency’ or ‘Social Withdrawal’ factors rather than core mood disturbance. In geriatric populations, subjective memory complaints and social withdrawal are frequently confounded by mild cognitive impairment, normal aging, or physical frailty that are highly prevalent regardless of depression status. Consequently, CTT-based scales that retain such items tend to exhibit reduced specificity due to elevated false-positive rates. Indeed, while Almeida’s CTT-based GDS10 [8] maintained high sensitivity (84.8%), its specificity (70.9%) was notably lower than our IRT-based version (84.0%), a pattern consistent with the inclusion of these cognitively confounded items.

By utilizing IRT discrimination parameters, we systematically filtered out these non-specific items. Items 12 (*a* = 0.70) and 14 (*a* = 0.88) exhibited notably low discrimination values, confirming their poor ability to differentiate depressed from non-depressed individuals. Through prioritizing items reflecting core dysphoria rather than cognitive or behavioral symptoms, GDS10-IRT achieves robust diagnostic equivalence to the full GDS30 despite having 33% fewer items than the GDS15, demonstrating that the widely accepted 15-item threshold was an artifact of CTT methodology rather than a true psychometric necessity.

Furthermore, the pattern of item exclusion across all abbreviated versions reinforces the need to minimize cognitive confounds. Items universally excluded from GDS15, GDS10, and GDS4 (e.g., Items 20, 26, 29, 30) generally reflect cognitive inefficiency or apathy rather than mood disturbance. For instance, Item 29 (‘ease of decision making’) exhibited the lowest discrimination parameter in our study (*a* = 0.20). The consistent rejection of these items across widely divergent methodologies—both CTT and IRT—confirms that cognitive symptoms function primarily as diagnostic noise in older adults. Additionally, items retained only in the GDS15 but dropped in all shorter versions (Items 7 and 23) demonstrated relatively modest discrimination (*a* ≈ 1.0–1.3), suggesting they offer marginal diagnostic value compared to the ‘elite’ items retained in the 10-item versions.

### 4.2. The Rediscovery of Item 16 and Cultural Context

Our identification of Item 16 (‘Do you often feel downhearted and blue?’) as the single most discriminating symptom (*a* = 2.53; AUC = 0.779) challenges both methodological conventions and cross-cultural assumptions. Previous cross-cultural research suggested that East Asian older adults might minimize direct expressions of sadness due to cultural stigma, with a tendency to suppress positive affect endorsement and show heightened interpersonal sensitivity [18]. Given these observations, one might expect somatic items to outperform mood items in Korean populations.

However, Item 16’s dominance contradicts this expectation and suggests that when queried directly about ‘downheartedness’, translated in Korean as ‘울적하고 침울하다’ (wool-juk-ha-go chim-wool-ha-da), capturing a culturally resonant blend of melancholy and lethargy, it functions as a trans-cultural core marker of depression. The Korean translation may possess particular salience because it employs indigenous emotional vocabulary rather than direct Western psychiatric terminology, potentially reducing cultural barriers to endorsement.

CTT methods likely excluded Item 16 from the GDS15 due to high multicollinearity with other mood items, misinterpreting its diagnostic potency as statistical redundancy. IRT analysis corrects this historical oversight by demonstrating that Item 16’s high correlation with depression status (precisely what makes it ‘redundant’ in CTT terms) is exactly what makes it the most powerful diagnostic anchor. This distinction between statistical redundancy (CTT perspective) and diagnostic information (IRT perspective) represents a fundamental methodological insight with implications beyond this specific scale.

### 4.3. Capturing Agitated Depression in Late Life

The GDS10-IRT’s inclusion of Item 11 (‘Do you often feel restless and fidgety?’) and Item 25 (‘Do you frequently feel like crying?’), both absent from the GDS15, reflects an important phenomenological consideration. Our previous factor analytic work classified these items under a distinct ‘Sad Mood and Agitation’ factor [15], distinguishing them from items measuring cognitive symptoms or social withdrawal.

Late-life depression frequently presents with agitation, irritability, and emotional lability rather than the classic retarded, anhedonic presentation more typical of younger adults. This ‘agitated depression’ phenotype is particularly common in the context of vascular pathology, white matter disease, and executive dysfunction—conditions highly prevalent in geriatric populations [19,20,21]. By retaining items that capture psychomotor agitation and emotional dysregulation, the GDS10-IRT may enhance detection of these non-melancholic depression presentations that might be missed by scales emphasizing only sadness or anhedonia.

Furthermore, four items included in GDS10-IRT but absent from GDS15 (Items 6, 11, 16, 25) collectively capture core dysphoria, restlessness, and emotional lability—symptom dimensions particularly relevant in Asian older adult populations where somatic and anxiety presentations of depression are common. This item configuration may explain the GDS10-IRT’s robust performance despite containing fewer items than GDS15.

### 4.4. Measurement Invariance and Clinical Implementation

DIF analysis revealed item-level variation across sex, age, and recruitment setting. Notably, women more readily endorsed emotional expression items (e.g., ‘feeling like crying’), consistent with known sex differences in depression phenomenology. Older adults more readily endorsed memory complaints and feelings of worthlessness, likely reflecting age-related concerns. Critically, despite item-level DIF, scale-level diagnostic performance remained equivalent across sex and age groups. This pattern of ‘compensatory DIF’—where items favoring one group are balanced by items favoring another—indicates that GDS10-IRT total scores can be validly compared across these subgroups without adjustment. However, the larger AUC difference by recruitment setting reflects true population differences in depression characteristics rather than measurement bias.

While ≥4 was identified as the optimal cut-off based on Youden’s J index, clinical context should guide threshold selection. In low-prevalence community screening, a higher cut-off (≥5) may be preferable to reduce false-positive burden. Conversely, in high-prevalence settings such as geriatric clinics or nursing homes (15–30%), refs. [22,23] a lower cut-off (≥3) maximizes sensitivity to minimize missed cases.

### 4.5. Limitations

Several limitations warrant consideration. First, the depression prevalence in our community sample (3.2% in KLOSCAD) is lower than typically reported in Western cohorts (8–15%). However, this class imbalance actually provides a more stringent test of specificity, as any scale maintaining high AUC under low-prevalence conditions demonstrates robust discriminative ability rather than inflated performance from base-rate effects. The consistent results across our community and clinical subsamples (prevalence 13.0%) further support generalizability across prevalence spectra. Regarding predictive values, while AUC demonstrated stability across development and validation sets (*p* = 0.584), the GDS10-IRT’s estimated positive predictive value (PPV) is approximately 14%—a common characteristic of screening tests in low-prevalence populations. Importantly, the high specificity ensures excellent negative predictive value (>99%), supporting efficient case identification. In higher-prevalence settings such as geriatric clinics (20%), PPV would increase to approximately 56%. Second, the prominence of Item 16 (‘downhearted and blue’) as the highest-discriminating symptom may appear to contradict observations that Asian populations preferentially report somatic over mood symptoms [15,24,25]. However, our finding suggests that when directly queried using culturally adapted instruments, Korean older adults do endorse core dysphoric symptoms. This challenges oversimplified assumptions about cultural differences in depression expression and underscores the importance of psychometrically validated translations. Third, while our findings derive from Korean older adults, the identification of ‘downheartedness’ and ‘emptiness’ as core discriminating features aligns with cross-cultural psychiatric consensus on depression phenomenology, suggesting these parameters likely translate to other populations. Nevertheless, external validation in Western and other Asian samples is warranted to confirm generalizability. Implementation in non-Korean populations should follow a staged validation approach, first testing the fit of Korean-derived IRT parameters before proceeding to population-specific recalibration if significant misfit is observed. Given that our items assess universal depression constructs, we anticipate reasonable cross-cultural stability, though cultural differences in emotional expression may require adjustment of specific parameters. Fourth, diagnostic assessments utilized DSM-IV criteria; however, given the substantial continuity in core depressive symptom definitions across DSM editions [26], we anticipate minimal impact on the scale’s applicability to current diagnostic practice. Finally, item selection focused exclusively on discrimination; future work might incorporate content validity considerations. Notably, the deliberate exclusion of cognitively confounded items (e.g., ‘memory problems’) may enhance the GDS10-IRT’s utility in cognitively impaired populations by focusing on core dysphoric symptoms rather than cognitive complaints that overlap with dementia. However, direct empirical validation is needed; future studies should examine GDS10-IRT performance across the cognitive spectrum, including MCI and mild-to-moderate dementia.

## 5. Conclusions

IRT-based item selection achieves GDS30 equivalent diagnostic performance with only 10 items (a 67% reduction) with stable cross-validation confirming generalizability. GDS10-IRT outperforms the GDS15 while using 33% fewer items. The highest-discriminating item (Item 16) is absent from GDS15, illustrating the cost of CTT-based abbreviation. These findings suggest that population-specific IRT optimization from complete item pools should become the standard approach for developing efficient, culturally sensitive screening instruments.

## Figures and Tables

**Table 1:** Demographic and clinical characteristics of the study participants.

Characteristics	All (N = 6525)	Datasets		
		Development (n = 3262)	Validation (n = 3263)	*p* *
Age, years	70.0 ± 6.7	70.0 ± 6.7	70.1 ± 6.6	0.247
Female	3711 (56.9)	1848 (56.7)	1863 (57.1)	0.872
Education, years	8.3 ± 5.3	8.2 ± 5.3	8.4 ± 5.3	0.118
Clinic sample	401 (6.1)	200 (6.1)	201 (6.2)	0.899
GDS, points	10.1 ± 6.6	10.0 ± 6.5	10.2 ± 6.7	0.194
Depressive Disorders ^a^	249 (3.8)	124 (3.8)	125 (3.8)	1.000
KLOSCAD sample	196 (3.2)	98 (3.2)	98 (3.2)	1.000
Clinic sample	53 (13.2)	26 (13.0)	27 (13.4)	0.940

Continuous variables are presented as mean (standard deviation) and categorical variables as number (%). GDS, 30-item original version of Geriatric Depression Scale; KLOSCAD, Korean Longitudinal Study on Cognitive Aging and Dementia. * Student *t*-test for continuous variables and chi-square test for categorical variables. ^a^ Major or minor depressive disorders according to DSM-IV criteria.

**Table 2:** Item-level psychometric properties, diagnostic utility, and composition across geriatric depression scale versions.

Items of Original GDS [3]	Abbreviated Versions				Statistics		
	GDS15 [6]	GDS4 [7]	GDS10 [7]	GDS10-IRT	*a* ^a^	*b* ^b^	AUC ^c^
**1. Satisfied with life?**	✓	✓	✓	✓	1.70	0.83	0.694
2. Dropped activities/interests?	✓		✓		0.78	−0.55	0.615
3. Feel that your life is empty?	✓	✓	✓		1.60	0.25	0.682
**4. Often get bored?**	✓		✓	✓	1.74	0.48	0.709
5. Hopeful about the future?					0.97	−0.30	0.607
**6. Bothered by thoughts?**				✓	1.62	0.78	0.669
7. Good spirits most of time?	✓				1.29	1.10	0.716
8. Something bad will happen?	✓		✓		1.28	0.97	0.657
9. Happy most of the time?	✓	✓	✓		1.55	0.85	0.704
**10. Feel helpless?**	✓		✓	✓	1.84	0.87	0.712
**11. Get restless and fidgety?**				✓	1.88	1.27	0.711
12. Prefer to stay at home?	✓		✓		0.61	1.42	0.657
13. Worry about future?					1.42	0.56	0.657
14. More memory problems?	✓		✓		0.90	1.51	0.654
15. Wonderful to be alive now?	✓	✓	✓		1.24	1.26	0.654
**16. Downhearted and blue?**				✓	2.47	0.60	0.744
**17. Feel pretty worthless?**	✓		✓	✓	1.93	0.92	0.707
18. Worry about the past?					1.53	1.33	0.667
19. Find life very exciting?					1.43	−0.07	0.671
20. Hard to start new projects?					0.65	−0.82	0.610
**21. Feel full of energy?**	✓		✓	✓	1.68	−0.07	0.695
**22. Situation is hopeless?**	✓		✓	✓	2.31	1.29	0.676
23. Others better off than you?	✓				1.03	1.08	0.653
24. Upset over little things?					1.59	0.63	0.696
**25. Frequently feel like crying?**				✓	2.29	1.20	0.738
26. Trouble concentrating?					1.06	0.80	0.679
27. Enjoy getting up in the morning?					0.84	1.80	0.671
28. Avoid social gatherings?					0.75	2.26	0.612
29. Easy to make decisions?					0.20	0.32	0.554
30. Mind as clear as it used to be?					0.86	−0.22	0.676

GDS, Geriatric Depression Scale; AUC, area under the curve. ^a^ Discrimination parameter estimated using the two-parameter logistic (2PL), indicating the item’s ability to differentiate between trait levels. ^b^ Difficulty parameter estimated using the two-parameter logistic (2PL), representing the threshold at which endorsement probability is 50%. ^c^ Calculated via receiver operating characteristic analysis, reflecting individual diagnostic utility. Bold font is used to denote items included in the GDS-10-IRT.

**Table 3:** Sequential item reduction analysis with cross-validation.

Scale	Development Set (n = 3262)			Validation Set (n = 3263)			Statistics	
	AUC (95% CI)	ΔAUC ^a^	*p* ^b^	AUC (95% CI)	ΔAUC ^a^	*p* ^b^	ΔAUC ^c^	*p* ^d^
GDS30 [3]	0.874 (0.844–0.903)	Ref.	-	0.883 (0.851–0.911)	Ref.	-	−0.009	0.673
GDS15 [6]	0.856 (0.826–0.888)	−0.018	0.012	0.859 (0.826–0.890)	−0.024	<0.001	−0.002	0.913
GDS10 [8]	0.846 (0.813–0.880)	−0.027	0.004	0.849 (0.817–0.880)	−0.034	<0.001	+0.002	0.925
IRT-based items								
10 items	0.859 (0.818–0.900)	+0.009	0.312	0.856 (0.809–0.895)	+0.010	0.396	−0.007	0.584
9 items	0.849 (0.817–0.881)	+0.017	0.003	0.877 (0.848–0.903)	+0.006	0.298	−0.028	0.206
8 items	0.841 (0.798–0.884)	+0.027	0.012	0.833 (0.788–0.877)	+0.029	0.016	−0.028	0.498
7 items	0.829 (0.785–0.873)	+0.039	0.001	0.822 (0.777–0.874)	+0.040	0.001	−0.007	0.562

AUC, area under the curve; CI, confidence interval; GDS, Geriatric Depression Scale; IRT, Item Response Theory. ^a^ Absolute difference in AUC values compared to the AUC of GDS30 in each dataset. ^b^ Comparing the AUC of each short forms compared to the AUC of GDS30 by the DeLong test [16] in each dataset. ^c^ Absolute difference in AUC values between the development and validation datasets. ^d^ Comparing the AUC between the development and validation sets using the Z-test for independent samples (Hanley & McNeil method) [17].

**Table 4:** Comparisons of Screening Performance and Efficiency.

Scale	Development Set			Validation Set			Statistics					
	Sensitivity	Specificity	Efficiency ^a^	Sensitivity	Specificity	Efficiency ^a^	Sensitivity		Specificity		Efficiency ^a^	
							*p* ^b^	*p* ^c^	*p* ^b^	***p* ** ^c^	***p* ** ^d^	***p* ** ^e^
All												
GDS30 [3]	80.6	82.0	0.029	83.2	80.4	0.029	Ref.	0.877	Ref.	0.535	Ref.	0.562
GDS15 [6]	83.1	73.6	0.057	73.6	84.4	0.056	0.286	0.881	0.684	0.678	<0.001	0.913
GDS10 [8]	81.5	72.2	0.085	84.8	70.9	0.085	0.754	0.481	<0.001	0.229	<0.001	0.925
GDS10-IRT	81.5	76.7	0.097	80.0	84.0	0.097	0.168	0.883	0.528	0.827	<0.001	0.206
KLOSCAD												
GDS30 [3]	77.7	83.2	0.0285	76.6	82.7	0.0282	Ref.	0.856	Ref.	0.614	Ref.	0.689
GDS15 [6]	74.5	82.0	0.0558	73.4	82.0	0.0553	0.257	0.865	0.725	0.702	<0.001	0.734
GDS10 [8]	92.9	61.2	0.0849	93.9	60.1	0.0859	0.002	0.774	<0.001	0.366	<0.001	0.682
GDS10-IRT	73.4	80.4	0.0928	72.3	81.5	0.0918	0.134	0.871	0.561	0.785	<0.001	0.562
Clinic												
GDS30 [3]	83.7	78.9	0.0298	83.3	83.2	0.0295	Ref.	0.948	Ref.	0.871	Ref.	0.724
GDS15 [6]	81.4	83.2	0.0125	76.7	77.5	0.0120	0.414	0.955	0.782	0.863	<0.001	0.768
GDS10 [8]	65.4	83.9	0.0798	55.6	84.5	0.0777	0.125	0.465	0.001	0.883	<0.001	0.770
GDS10-IRT	81.4	77.5	0.0972	83.3	77.2	0.0981	0.480	0.798	0.617	0.934	<0.001	0.618

GDS, Geriatric Depression Scale. ^a^ AUC/number of items, representing diagnostic accuracy per item. Higher values indicate greater screening efficiency. ^b^ Compared to the GDS30 by McNemar test. ^c^ Comparison between the development and validation datasets by chi square test. ^d^ Compared to the GDS30 by bootstrap percentile method (1000 iterations). ^e^ Comparison between the development and validation datasets by z test based on bootstrap standard errors.

**Table 5:** Differential Item Functioning (DIF) and Scale-Level Performance.

Item	Content	Sex		Age Group		Setting	
		|ΔMH|	ETS	|ΔMH|	ETS	|ΔMH|	ETS
Item-Level DIF ^a^ by Sex, Age Group, and Recruitment Setting							
GDS01	Satisfied with life (R)	0.81	A	0.23	A	0.66	A
GDS04	Often get bored	0.41	A	0.85	A	0.84	A
GDS06	Afraid something bad will happen	1.35	B	0.07	A	1.34	B
GDS10	More memory problems	0.99	A	1.51	C	1.17	B
GDS11	Wonderful to be alive (R)	1.24	B	0.04	A	1.35	B
GDS16	Downhearted and blue	1.18	B	0.23	A	1.69	C
GDS17	Feel worthless	0.44	A	1.53	C	1.49	B
GDS21	Full of energy (R)	0.97	A	0.91	A	1.47	B
GDS22	Situation is hopeless	0.85	A	1.06	B	1.06	B
GDS25	Feel like crying	2.27	C	0.05	A	1.58	C
Scale-Level Differential Test Functioning							
Comparison		AUC (95% CI)				ΔAUC	
(Group 1, Group 2)		Group 1		Group 2			
Sex (male, female)		0.856 (0.786–0.925)		0.817 (0.778–0.856)		0.039	
Age (<75 years, ≥75 years)		0.839 (0.801–0.878)		0.832 (0.762–0.902)		0.007	
Setting (community, clinic)		0.861 (0.827–0.895)		0.695 (0.597–0.794)		0.166	

AUC, Area Under the Curve; CI, confidence interval; DIF, Differential Item Functioning; ΔMH, Mantel-Haenszel delta; ETS, Educational Testing Service; GDS, Geriatric Depression Scale; (R) = Reverse-scored item. ^a^ DIF was assessed using the Mantel-Haenszel (MH) procedure. ETS classification: A = negligible (|ΔMH| < 1.0); B = moderate (1.0 ≤ |ΔMH| < 1.5 with *p* < 0.05); C = large (|ΔMH| ≥ 1.5 with *p* < 0.05).

## Data Availability

The data that support the findings of this study are available from the corresponding author upon reasonable request.
